# Factors influencing breastfeeding failure in mothers with hepatitis B infection: a qualitative study

**DOI:** 10.3389/fnut.2026.1839059

**Published:** 2026-07-02

**Authors:** Xin Jiang, Hui Jiang, Rong Huang

**Affiliations:** Nursing Department, Shanghai Key Laboratory of Maternal Fetal Medicine, Shanghai Institute of Maternal Fetal Medicine and Gynecologic Oncology, Shanghai First Maternity and Infant Hospital, School of Medicine, Tongji University, Shanghai, China

**Keywords:** breastfeeding failure, hepatitis B virus, mothers, public health, qualitative research

## Abstract

**Purpose:**

To explore the factors contributing to breastfeeding failure among mothers infected with the hepatitis B virus (HBV) from the perspectives of stress and decision-making, with a specific focus on psychological factors.

**Design:**

A qualitative, descriptive design using semi-structured interviews.

**Methods:**

Purposive sampling recruited 21 HBV-positive mothers at Shanghai First Maternity and Infant Hospital between February 1 and May 10, 2023. Data collection continued until thematic saturation. Interviews were audio-recorded, transcribed verbatim, and analyzed via thematic analysis with NVivo 12.6.1 software. Measures to ensure data trustworthiness included member checking, peer debriefing, and investigator triangulation.

**Results:**

Three participants withdrew before completion, resulting in 18 final interviews. Mean maternal age was 32.28 ± 4.80 years; 56% delivered by cesarean section, 67% had female infants. Five mothers were HBsAg-positive only, 13 were both HBsAg- and HBeAg-positive. Four mothers used partial breastfeeding, 13 formula feeding, only one practiced exclusive breastfeeding during hospitalization. Four core themes emerged: “Inadequate Knowledge of HBV Transmission Routes, Breastfeeding Benefits, and Resultant Decision-Making Uncertainty” (Cognitive Knowledge Deficits); Persistent Fear of Getting An Infant Infected With HBV, Despite Reassurance and Medical Information From Healthcare Providers (Emotional/psychological concerns); Practical Technical Difficulties in Breastfeeding, and Lactation-related Complications Compromising Feeding Sustainability (Persistent physiological and technical breastfeeding barriers); Family Members’ Opposition to Breastfeeding that Increases Above the Maternal Feeding Intent and Directly Affects Infant Feeding Outcome (Family opposition and maternal intent deviate from infant feeding outcomes).

**Conclusion:**

Breastfeeding among HBV-positive mothers is influenced by multiple factors including knowledge gaps, psychological concerns, physical challenges, and social pressures. Interventions should focus on improving knowledge, reducing psychological stress, enhancing technical support and engaging family members to promote breastfeeding continuation.

**Impact:**

The study identifies HBV-positive mothers’ breastfeeding barriers. It offers evidence for healthcare institutions’ targeted strategies and helps improve breastfeeding rates and infant health.

## 1 Introduction

Chronic hepatitis B (CHB) is one of the most important global health issues. Hepatitis B virus (HBV) is an enveloped hepatotropic DNA virus that infects only primates (1). Hepatitis B virus causes considerable morbidity and mortality worldwide and is the major cause of cirrhosis and liver cancer all over the world (2). Worldwide, about one-third of the population has had hepatitis B virus infection and 257 million people have chronic HBV infection (3). Each year 887, 000 people die because of hepatitis B virus infection, mainly due to cirrhosis and hepatocellular carcinoma (4). Notably, HBV mother-to-child transmission not only increases the global public health burden of CHB by enlarging the infected population size, but also results in the long-term health inequality in different generations, generating the inequality of maternal and child health, that is, children infected from mother-to-child face a higher lifetime risk for chronic liver diseases, and mothers with HBV infection also have extra health or social burdens, widening the health gap between the two vulnerable populations and the general population, in terms of health.

As the HBsAg prevalence rate in China is 6.89%, mother-to-child transmission (MTCT) of HBV during intrapartum period is the most prevalent route of HBV transmission in China (5). MTCT in high prevalence areas is responsible for >50% of chronic HBV infections and 1–9% of newborns of parents with chronic HBV get HBV infection in early life (6, 7). This persistent MTCT burden places a heavy healthcare burden on the healthcare system of China including long-term costs of treatment for liver disease, disease prevention and health maintenance, and maternal and child health gap is still persistent. At present, several clinical guidelines clearly explain that direct breastfeeding is possible for women with hepatitis B, irrespective of Hepatitis B e Antigen (HBeAg) status (8–11), and has no additional risk for HBV infection of newborn (12). However, the breastfeeding rate among HBV-infected mothers remains low in clinical practice, frequent breastfeeding obstacles and breastfeeding cessation even happened. The low breastfeeding rate deprives newborn babies of the nutritional and immune benefits of breast milk, and, at the same time, makes it impossible to make use of a simple and low-cost intervention so as to narrow the gap in health, worsening the public health impact of HBV MTCT.

Existing studies have demonstrated the multifactorial nature of breastfeeding barriers in this population: Zhang et al. (13) indicated that maternal cognitive biases regarding HBV MTCT routes are core psychological barriers; a cohort study (14) showed that irregular, inconsistent breastfeeding guidance from medical staff easily triggers maternal feeding anxiety, which in turn leads to breastfeeding cessation; additionally, Mokaya et al. (15) found that insufficient family support, social stigma toward HBV-infected individuals, and maternal concerns about the impact of their condition on milk quality are all important factors hindering the sustainability of breastfeeding. Meanwhile, several studies (16, 17) have confirmed a series of effective measures for improving the aforementioned predicament: an intervention study showed that systematic health education on breastfeeding including transmission mechanisms and protective measures for HBV-infected pregnant women can increase breastfeeding willingness by more than 40%; Huang et al. (18) pointed out that establishing a specialized medical team to provide individualized feeding guidance, combined with family support interventions, can significantly reduce the breastfeeding cessation rate; furthermore, standardized administration of hepatitis B immunoglobulin and hepatitis B vaccination to newborns, as basic protective measures, also provides important guarantees for eliminating maternal feeding concerns and maintaining breastfeeding (19). Addressing these barriers and promoting breastfeeding among HBV-infected mothers is crucial for reducing the public health burden of HBV MTCT and advancing maternal and child health equity.

Albeit the aforementioned guidelines have confirmed breastfeeding is safe in HBV positive mothers and those relevant studies have first explored the breastfeeding barrier and influencing factors, most studies are quantitative research and lack detailed consideration on mothers’ subjective experience, mental activities and breastfeeding decisions; there is a discrepancy between guidelines and clinical practice: some HBV positive mothers give up breastfeeding because of complicated psychological and social reasons even though they knew it was safe, and their subjective mental logic and experienced pain points have not been fully portrayed (19). The existing gap handicaps targeted interventions in public health research, which further hinders endeavors to diminish the burden of HBV MTCT and promote maternal and child health equity.

We adopted a phenomenological perspective to explore the lived experiences and internal cognitions of HBV-infected mothers during breastfeeding. On the basis of standard thematic analysis procedures, semi-structured in-depth interviews were performed to collect original data concerning these mothers’ subjective feelings, cognitive perceptions, and behavioral motivations related to breastfeeding (20–22). Strictly following standardized analytical procedures including data transcription, open coding, axial coding, and core theme extraction and summarization, this study systematically investigated the multidimensional psychological, social, and clinical factors as well as the underlying mechanisms affecting breastfeeding practices among HBV-infected mothers. By integrating phenomenological ideas with standardized thematic analysis, the research paradigm effectively restores participants’ true perceptions and decision-making processes. The findings provide empirical evidence for developing precise and individualized clinical intervention schemes, which offers practical value for reducing the disease burden of HBV mother-to-child transmission and advancing health equity.

Therefore, this study aims to fill the existing research gap by identifying key factors hindering breastfeeding among HBV-infected mothers. The findings will provide evidence to optimize clinical interventions and correct common cognitive biases, thereby improving breastfeeding rates and reducing the risk of HBV mother-to-child transmission. By exploring mothers’ subjective experiences, this study also helps inform public health strategies to ensure equitable breastfeeding support for vulnerable mothers and promote maternal and child health equity.

## 2 Materials and methods

### 2.1 Study sample and recruitment

We adopted a thematic analysis approach, which aims to explore the subjective experiences, perceptions, and feelings of HBV-positive postpartum mothers regarding breastfeeding in depth. Data were collected through one-on-one semi-structured in-depth interviews, a method suitable for capturing rich, context-specific narratives of individual experiences (23). During the research protocol development phase, two senior obstetricians with extensive experience in hepatitis B virus (HBV) mother-to-child transmission prevention (PMTCT) were consulted to ensure the scientificity and feasibility of the study design. Subsequently, the interview protocol was further refined by soliciting opinions from two obstetric nurse specialists, all of whom had professional experience in HBV PMTCT, to enhance the comprehensiveness and pertinence of interview content.

We recruited full-term postpartum mothers with HBV infection from February 1 to May 10, 2023 at Shanghai First Maternity and Infant Hospital, a tertiary maternal and child health institution specializing in HBV mother-to-child transmission prevention. Given its large number of HBV-positive pregnant and postpartum women, the hospital guaranteed adequate participant recruitment and ensured the research results were representative of clinical practice in East China. Participants were selected via purposive sampling according to unified inclusion and exclusion criteria. Interviews were conducted until data saturation was reached with no new themes appearing. Initially, 21 mothers were enrolled, while 3 dropped out, leaving 18 participants for final analysis. All participants were assigned pseudonyms M1 to M18 to protect personal privacy.

#### 2.1.1 Inclusion and exclusion criteria

Inclusion criteria: (1) Full-term postpartum mothers (gestational age ≥ 37 weeks) diagnosed with HBV infection (defined as positive for HBsAg for more than 6 months or positive for HBsAg and HBV DNA at the same time); (2) Postpartum period within 3 days; (3) Able to communicate fluently in Mandarin or Shanghainese to complete the interview; (4) Voluntarily participate in the study and sign the informed consent form; (5) Covering newborns of different genders and different socio-economic levels (socio-economic level was evaluated based on monthly family income, educational background, and occupation, including low, medium, and high levels) to ensure the diversity of sample and avoid selection bias.

Exclusion criteria: (1) Mothers with other severe infectious diseases or serious maternal complications; (2) Mothers with mental illness or cognitive impairment who cannot express their experiences clearly; (3) Newborns with severe congenital malformations or diseases that affect breastfeeding; (4) mothers refusing or prohibited from breastfeeding.

### 2.2 Interview guide

This interview aimed to explore the breastfeeding experiences and subjective feelings of HBV-positive mothers. Based on the research objectives, five open-ended interview questions were designed: (1) Please describe your understanding of HBV transmission and the benefits of breastfeeding, as well as your concerns about breastfeeding. (2) Share your feelings during breastfeeding and how these emotions affected your feeding persistence. (3) Please state the difficulties you faced while attempting to breastfeed and how these challenges affected you. (4) What attitudes did your family hold toward your breastfeeding, and how did these attitudes influence your feeding decisions? (5) Do you have any other feelings or experiences about breastfeeding to share?

### 2.3 Data collection procedures

Before the interview, each potential participant was fully informed of the study purpose, interview duration (approximately 30–40 min), data collection methods, potential risks, and rights, including the right to withdraw at any time without penalty. Written informed consent was obtained from all participants, including specific consent for audio recording. Interviews were conducted in a private, undisturbed consulting room of the hospital during the participants’ hospitalization period to avoid external interference and create a safe and trusting communication environment.

During the interview, participants were allowed to speak freely without any guidance or suggestive cues from researchers. They were encouraged to share their authentic breastfeeding experiences and inner feelings. Researchers recorded key interview information and monitored participants’ physical and emotional conditions. If any participant felt emotionally uncomfortable during the conversation, the interview would be terminated immediately. Researchers provided emotional comfort and reminded participants of their right to withdraw from the study at any time.

### 2.4 Data analysis

We adopted the thematic analysis method to process qualitative data. First, all audio and video records of interviews were fully transcribed into written texts within 24 h after each interview to minimize information loss. The transcribed texts were cross-checked by two researchers against the original audio-video materials and interview notes to guarantee data accuracy. Subsequently, two trained members of the research team independently coded the interview transcripts using NVivo analysis software (Version 12.6.1). Initially, the two researchers separately coded and analyzed two randomly selected transcripts to develop a preliminary coding manual with initial codes and corresponding definitions. They then held discussions to compare and reconcile coding discrepancies, revise and standardize the coding manual, and reach a consensus on ambiguous coding contents. This iterative process was implemented throughout the analysis: new codes were continuously supplemented to the coding manual and existing codes were refined and optimized with the analysis of additional transcripts until thematic saturation was achieved. Saturation was confirmed by the entire research team when no new codes or sub-themes emerged in successive rounds of coding for newly supplemented interview texts.

To ensure the rigor, credibility, and validity of the study, we selected 10 randomly selected participants reviewed theme summaries and their quotes, providing feedback to revise themes/codes and ensure alignment with their experiences. Then, 2 external researchers were invited to review the study materials, providing feedback to refine codes/themes and reduce researcher bias. Researchers with diverse expertise including obstetric nurse, nurse manager, public health researcher, participated in data collection and analysis to validate findings. Last, the team members maintained reflexive journals and held meetings to identify and mitigate bias from personal/professional backgrounds.

### 2.5 Quality control

Participants were fully informed of the study’s objectives and assigned pseudonyms (M1–M18) for privacy protection. Interviewers had no prior professional or personal relationship with participants; the lead interviewer had over 6 months of obstetric clinical experience and expertise in HBV maternal-newborn nursing, and all team members received centralized training on study protocols, interview skills and ethical norms pre-study. Researchers identified themselves only as clinical research staff during interviews, using professional knowledge to relieve participants’ anxiety and facilitate open sharing.

To enhance study trustworthiness, key strategies were adopted: two researchers independently analyzed data in separate locations to avoid mutual influence, with coding/interpretive inconsistencies cross-checked against original recordings/transcripts and member checking with participants if needed; standardized team training minimized researcher bias, and the lead interviewer’s professional background fostered empathetic, trusting communication with participants; strict ethical measures (comprehensive informed consent with unconditional withdrawal rights, pseudonymization, secure data storage) and full transparency of research procedures further enhanced participants’ sense of security and the authenticity of shared information.

### 2.6 Ethical considerations

This study involving human participants was conducted in strict compliance with the principles of the Declaration of Helsinki and the ethical guidelines for medical research involving human subjects in China. The study protocol was reviewed and approved by the Ethics Committee of Shanghai First Maternity and Infant Hospital (Approval Number: KS24123). All participants provided written informed consent or verbal consent, and the entire research process complied with relevant national ethical norms.

## 3 Results

Initially, 21 women agreed to participate; three withdrew before completion, resulting in 18 final interviews. A total of 18 HBV-positive mothers were included in the final analysis. The mean age was 32.28 ± 4.80 years (range: 25–42 years). The majority were clerks (10/18, 55.6%), followed by unemployed women (3/18, 16.7%) and teachers (2/18, 11.1%), with other occupations (3/18, 16.7%). Ten mothers delivered by cesarean section, and 8 by vaginal delivery. Twelve infants were female and 6 were male. Five mothers were HBsAg-positive only, while 13 were both HBsAg- and HBeAg-positive. During hospitalization, 1 (5.6%) mother practiced exclusive breastfeeding, 4 (22.2%) used partial feeding, and 13 (72.2%) adopted formula feeding. The data can be found in Table 1.

**Table 1:** Demographic characteristics of the participants.

ID	Age	Occupation	Delivery mode	Baby sex	HBV results	Breastfeeding experience
1	30	Clerk	Cesarean section	Female	HBsAg(+)/HBeAg(+)	Formula
2	30	Clerk	Vaginal delivery	Female	HBsAg(+)HBeAg(+)	Formula
3	30	Clerk	Vaginal delivery	Female	HBsAg(+)/HBeAg(+)	Formula
4	34	Teacher	Cesarean section	Female	HBsAg(+)	Partial
5	42	Clerk	Cesarean section	Male	HBsAg(+)	Breastfeeding
6	40	None	Cesarean section	Female	HBsAg(+)/HBeAg(+)	Formula
7	36	None	Vaginal delivery	Female	HBsAg(+)	Formula
8	38	Doctor	Vaginal delivery	Female	HBsAg(+)/HBeAg(+)	Formula
9	26	Lawyer	Vaginal delivery	Female	HBsAg(+)/HBeAg(+)	Formula
10	25	Clerk	Cesarean section	Male	HBsAg(+)	Formula
11	28	Teacher	Vaginal delivery	Female	HBsAg(+)/HBeAg(+)	Formula
12	29	Clerk	Cesarean section	Male	HBsAg(+)/HBeAg(+)	Formula
13	30	Clerk	Cesarean section	Female	HBsAg(+)	Partial
14	33	Clerk	Cesarean section	Male	HBsAg(+)/HBeAg(+)	Formula
15	34	None	Vaginal delivery	Male	HBsAg(+)/HBeAg(+)	Formula
16	37	Clerk	Cesarean section	Female	HBsAg(+)/HBeAg(+)	Partial
17	28	Clerk	Cesarean section	Male	HBsAg(+)/HBeAg(+)	Formula
18	31	Engineer	Vaginal delivery	Female	HBsAg(+)/HBeAg(+)	Partial

### 3.1 Theme 1: “inadequate knowledge of HBV transmission routes, breastfeeding benefits, and resultant decision-making uncertainty” (cognitive knowledge deficits)

Many HBV-positive mothers demonstrated limited understanding of breastfeeding safety and the prevention of mother-to-child transmission (PMTCT). This knowledge deficit extended to their own disease status and the benefits of breastfeeding, creating uncertainty that often deterred breastfeeding initiation. Misconceptions about potential viral transmission through breast milk further reinforced decisions to avoid breastfeeding despite medical reassurance.

*Case M2:* “Hepatitis B can be transmitted and it is hard to eradicate.” *Case M5:* “The virus can be controlled with medication but may come back in the future.” *Case M12:* “The Hepatitis B virus may even be found in breast milk. If my baby drinks my breast milk, he will contract Hepatitis B. He is so young. How can I be so foolish?” “The patients in the same ward all refused, so I chose to feed the baby with formula milk for safety.” *Case M6:* “I was not clear whether I could breastfeed or not and I never asked the doctor.” *Case M11:* “Breastfeeding is like formula milk. Nowadays, there are lots of formula milk.” “Our first baby was born on formula milk and has become very strong; we have no problem with formula now.”

### 3.2 Theme 2: persistent fear of getting an infant infected with HBV, despite reassurance and medical information from healthcare providers (emotional/psychological concerns)

Despite reassurance from healthcare providers and the preventive strategies set in place, many HBV-positive mothers did not feel relieved of their fear of infecting their newborn infant with HBV. This fear was often a belief that the child was unsafe for breastfeeding, which itself bred more fear and refusal to breastfeed. Mother HBV-positive mothers’ fear was further exacerbated by their anxiety for their child’s future health and some opted for formula feeds because that was seen as a “safer” alternative option.

*Case M17:* “I was extremely worried about the potential consequences. Even though the doctor explained the low transmission risk after vaccination, I couldn’t shake the fear that my baby might become infected. This made it difficult for me to consider breastfeeding.” *Case M12:* “The medical staff have popularized relevant professional knowledge many times and told me that breastfeeding is safe after standardized prevention. However, as a mother, I cannot tolerate any risk to my baby’s health. The fear of causing lifelong HBV infection to my child has always haunted me, so I finally chose to give up breastfeeding to avoid all possible risks.”

### 3.3 Theme 3: practical technical difficulties in breastfeeding, and lactation-related complications compromising feeding sustainability (persistent physiological and technical breastfeeding barriers)

Technical difficulties and physiological problems were formidable obstacles to breastfeeding continuation. Many mothers had problems with how to latch properly and with their breastfeeding positions, often with painful complications, including mastitis and nipple cracking. Nevertheless, anxiety and low mood, psychological difficulties, and poor nutritional intake contributed to poor milk supplies. Collectively, these associated physical and emotional difficulties generated a cycle of pain and frustration that often led to premature abandonment of breastfeeding by HBV-infected mothers.

*Case M9:* “I had plenty of breast milk, but my child would not breastfeed and couldn’t fully empty my breasts on time.” *Case M15:* “My breasts were huge, and I tried using a breast pump and hand pump to express breast milk, but that did not take away my pain.” “The pain was so unbearable that I had fever, and I didn’t breastfeed anymore.” *Case M17:* “My breasts exploded when I gave birth to my child, and my nurse prompted me to breastfeed my baby for a few days.” *Case M13:* “I used a protective nipple shield to protect my nipples from being nibbled.” *Case M18:* “But the baby did not want to suck at the place after birth, I was in bad mood because of some disputes in the family; so my supply of breast milk reduced. Eventually I reached the point of deciding to stop breastfeeding.” Case M10: “My baby cried a lot at night, so I gave the baby a pacifier and kept her from disturbing the rest of the family members.”

### 3.4 Theme 4: family members’ opposition to breastfeeding that increases above the maternal feeding intent and directly affects infant feeding outcome (family opposition and maternal intent deviate from infant feeding outcomes)

Opposition from the woman’s spouse or family members often proved to be the proverbial straw that broke the camel’s back for breastfeeding. Even if the healthcare professional confirmed breastfeeding was safe for women infected with HBV, many mothers were still unsure. Such hesitation left women particularly vulnerable to family views. Relatives worry about potential virus transmission, which makes them prefer infant formula over breastfeeding. To avoid turmoil in the household and avoid being put to shame, many mothers eventually relented to their families and opted for formula feeding.

*Case M3:*“I wish to breastfeed. I know that breastfeeding is good for the baby and the mother, but the baby’s father does not like it. I know breastfeeding is beneficial to both the baby and the mother, but the father has a different idea. He forced me to feed artificially.” Case M2: “I want to feed my child by breast-feeding, but my husband feels breastfeeding is bad for the baby.” Case M18: “I breastfed for 2 days. But after my in-laws come to take care of me, they do not want me to breastfeed.” I told her breast-feeding is safe for both mother and baby and baby has antibodies to Hepatitis B, and I even asked doctor to explain to her. But she still strongly opposes breastfeeding. Finally I gave up breastfeeding.”

## 4 Discussion

### 4.1 The current situation of breastfeeding among HBV-positive mothers

Although global evidence consistently indicates that breastfeeding does not increase the risk of mother-to-child transmission (MTCT) of hepatitis B virus (HBV) when timely immunoprophylaxis measures are administered, a survey (24) in a multicenter cross-sectional study of 602 HBV-infected mothers investigated at 42 days postpartum, any breastfeeding was reported by 45.85% and exclusive breastfeeding by only 26.91%, both markedly lower than the contemporaneous national averages of 60% initiation and 40% exclusivity. Subgroup multivariate regression analyses derived from that cross-sectional dataset (24) further showed that fear of mother-to-child transmission (69.33%) and family opposition (35.58%) as the principal reasons for breastfeeding cessation.

Qualitative data further elucidated that this gap in breastfeeding rates is not solely attributed to insufficient knowledge regarding HBV and breastfeeding. Instead, it is driven by several intertwined and mutually reinforcing factors: prevalent folk misconceptions that “breast milk contains transmissible HBV,” the risk-averse decision-making power of grandmothers (who often exert significant influence on infant feeding choices in Chinese families), concerns about the potential transfer of antiviral medications through breast milk, inconsistent advice across different departments within hospitals, and the lack of sustained breastfeeding support following discharge from medical facilities.

Notably, these barriers mirror those reported in studies conducted in Nigeria (25), and other countries with high HBV prevalence, suggesting that such multi-faceted challenges are not unique to China but rather represent a global issue in maternal and child healthcare for HBV-positive populations. This convergence of findings underscores the urgent need for comprehensive, multi-level interventions that move beyond isolated patient education initiatives.

Instead, we propose for interventions to support mothers with hepatitis B virus (HBV) to incorporate specific solutions for addressing each barrier collectively: introducing grandmother-inclusive counseling sessions for mother to be consistent on her family’s perception of breastfeeding safety, disseminating the safety information based on pharmacokinetic evidence to alleviate their worries about transmission of antiviral drugs during breastfeeding, creating common clinical management pathways marked on the electronic medical records (EMR) system so that patients can receive a consistent counseling and advice during stay at different hospital departments, and offering the infant health-promoting post-discharge follow-up services via telehealth platforms offered by the International Board-Certified Lactation Consultant (IBCLC). Such coordinated effort is needed to overcome the barriers and enhance the breastfeeding intention of the mothers with HBV while protecting infant’s health.

Healthcare professionals play a critical and irreplaceable role in supporting successful breastfeeding among HBV positive mothers. First and foremost, as authoritative information providers, they should offer accurate, consistent, evidence based explanations of HBV transmission, breastfeeding safety, and standard neonatal immunoprophylaxis, so as to correct long standing misconceptions and reduce unnecessary maternal anxiety. Second, clinicians and certified lactation specialists can provide hands-on, personalized guidance covering effective positioning, proper latching, milk expression, and safe management of common lactation complications such as engorgement and nipple pain, thereby helping mothers sustain breastfeeding. Last, healthcare providers also act as family educators and communication facilitators, helping partners and grandparents understand the scientific evidence for breastfeeding safety. Such family-centered support can alleviate unfounded fears and reduce avoidable family resistance, creating a more supportive environment for breastfeeding continuation.

### 4.2 Psychological stress and negative emotions in HBV-positive mothers: urgent attention needed

Interviews revealed that HBV-positive mothers experienced heightened psychological stress influenced by multiple factors. The chronic, infectious nature of HBV, unpredictable disease progression, varied symptoms, medication side effects, and uncertain prognosis collectively contribute to reduced quality of life affecting over 80% of patients (26).

HBV-infected women, facing the double dilemma of dealing with their disease and pregnancy, labor and postpartum, have concerns about transmission of the virus to family members or newborns during blood, sexual contact or mother-to-child route, which induces anxiety and depression. Pregnancy additionally aggravates these emotions (27). Anxiety and stress associated with HBV not only influence maternal mental state, but also disease control, postpartum recovery, lactation and its promotion. Interventions are necessary and specific, including: education to correct misconceptions regarding transmission, based on scientific evidence; counseling, information, emotional support services; self-care guidance for HBV-positive mothers; peer guidance based on positive breastfeeding experience sharing; family involvement in sharing self-care experience and supporting the mother emotionally. All of the above can provide knowledge and emotional care for a favorable environment that supports breastfeeding and protects the mental health of women with HBV.

### 4.3 Publicizing knowledge and providing guidance on breastfeeding for mothers with hepatitis B

The survey results show that the knowledge rate of pregnant women with hepatitis B about the hepatitis B disease is low (28). In this interview, the main area where the lack of knowledge was observed among hepatitis B mothers was in the areas of PMTCT (Prevention of Mother-to-Child Transmission) and breastfeeding. Many mothers could not clearly distinguish the specific measures of PMTCT or confirm whether breastfeeding is safe after standard prophylaxis. This uncertainty makes mothers prefer safer feeding methods, such as artificial feeding. Meanwhile, inadequate knowledge of the benefits of breastfeeding, poor breastfeeding techniques resulting in engorgement, insufficient milk supply, and cracked nipples. Most of them are unaware that breastfeeding can enhance infants’ resistance to infections and promote maternal uterine recovery. Even those who attempt to breastfeed often give up prematurely due to not knowing how to relieve engorgement or improve milk supply. Additionally, the lack of professional guidance on addressing these practical problems further exacerbates their difficulties in sustaining breastfeeding.

### 4.4 Knowledge deficits and the need for targeted breastfeeding education

Survey findings indicated that pregnant women with hepatitis B demonstrated limited understanding of their disease (29). In our interviews, knowledge gaps were particularly evident regarding mother-to-child transmission (PMTCT) and breastfeeding. This uncertainty frequently led mothers to opt for formula feeding, which they perceived as “safer.” Deficiencies in knowledge extended beyond breastfeeding benefits to practical skills, contributing to problems such as engorgement, insufficient milk supply, and nipple cracks. These challenges often stemmed from inadequate prenatal education, misconceptions about postpartum physiology, difficulties adapting to the maternal role, and failure to seek timely professional assistance when breastfeeding problems arose. To address these barriers, breastfeeding education for HBV-positive mothers should use clear, accessible language tailored to individual educational backgrounds, combine theoretical knowledge with practical demonstrations, include multiple follow-up visits to correct improper techniques, and specifically emphasize breastfeeding safety when infants receive appropriate immunoprophylaxis. This comprehensive educational approach can empower HBV-positive mothers with both the knowledge and skills needed to successfully initiate and maintain breastfeeding while alleviating unnecessary transmission concerns.

### 4.5 The family support system for mothers with hepatitis B: an urgent priority

The postpartum period is characterized by reduced social engagement, making strong support systems particularly crucial for new mothers’ physical and psychological recovery. Compared to mothers without hepatitis B, women living with HBV face additional pressures due to concerns about mother-to-child transmission (MTCT), which often leads to an increase in their healthcare-seeking behavior. A positive family environment and robust social support can alleviate the burden associated with maternal HBV infection (30).

Family members’ attitudes significantly influence breastfeeding decisions. Caregivers often lack comprehensive knowledge about HBV MTCT and may harbor unfounded concerns, while breastfeeding decisions can evoke maternal guilt. We recommend that healthcare providers adopt a family-centered educational approach, involving spouses and key caregivers in counseling sessions so they can acquire relevant knowledge and skills together (31). Enhancing families’ understanding of HBV, the physiological changes of the perinatal period, and effective care strategies will enable caregivers to provide more informed and supportive assistance (32).

This inclusive approach addresses both individual and environmental factors, creating a supportive ecosystem that empowers HBV-positive mothers to make informed breastfeeding decisions while reducing family conflict and psychological stress.

## 5 Limitation

Our study used a semi-structured interview approach, and the study sample was purposive sampling of mothers infected with the hepatitis B virus (HBV) as target population. Although the purposive sampling approach allows detailed exposure of individual experiences, the study findings may lack generalizability. The sample size of our study is small and all respondents were recruited from one regional medical center. This sample may not represent the diversity of the HBV-infected mother population in different age groups, socioeconomic backgrounds, or geographical locations. Furthermore, this study only collected data from postpartum mothers and did not incorporate perspectives from family members, resulting in a one-sided understanding of breastfeeding barriers.

## 6 Strengths and novelties

This study focuses on the special group of postpartum mothers infected with hepatitis B virus (HBV) and has significant strengths and novelties in research perspective and methodology. Firstly, it accurately targets the core pain points of HBV-positive mothers in making breastfeeding decisions, adopting a thematic analysis combined with one-on-one semi-structured in-depth interviews. This method deeply explores the subjective experiences, perceptions and potential psychological activities of the participants, making up for the deficiency of quantitative research in capturing individual emotions and complex decision-making logic, and providing rich qualitative evidence for understanding the breastfeeding behavior of this group.

Secondly, the interview protocol was revised and optimized after extensive demonstration by obstetricians, clinical nurse specialists and psychologists. Meanwhile, the researchers have solid clinical experience in obstetrics and professional knowledge of nursing care for HBV-positive mothers and newborns, which enables them to quickly establish a trusting relationship with participants, effectively improve the authenticity and depth of interview data, and ensure the research quality.

Thirdly, the study considers dimensions such as the newborn’s gender and different socioeconomic levels in sample selection. The sample size is determined based on the principle of data saturation, and a series of quality control measures such as independent coding by two researchers and cross-validation are adopted to ensure the rigor and credibility of the research results. These provide targeted references for clinical nursing interventions in the field of HBV mother-to-child transmission prevention (PMTCT).

The novelty of this study lies in breaking through the traditional focus on “whether breastfeeding is feasible” for HBV-positive mothers, and instead focusing on the decision-making dilemmas, emotional experiences and support needs of this group during breastfeeding, especially paying attention to potential hidden influencing factors. It provides a new research perspective for formulating personalized breastfeeding support programs and optimizing HBV PMTCT nursing services in clinical practice, thereby enriching the qualitative research achievements in this field.

## 7 Conclusion

We explored breastfeeding barriers among HBV-positive mothers through semi-structured interviews with 18 participants. The findings identified four major obstacles: cognitive knowledge deficits; emotional/psychological concerns; physical and technical difficulties with breastfeeding; and family opposition influenced by misconceptions. Breastfeeding safety and HBV transmission risk with adequate neonatal immunoprophylaxis have been well validated in existing guidelines and prior literature. The study recommend comprehensive interventions addressing knowledge gaps, psychological support needs, technical breastfeeding skills, and family involvement to improve breastfeeding rates among this population and provided valuable insights for developing family-centered educational programs and support systems that empower HBV-positive mothers to make informed breastfeeding decisions.

## Data Availability

The original contributions presented in the study are included in the article/supplementary material, further inquiries can be directed to the corresponding authors.
